# Defining *Paenibacillus azoreducens* (P8) and *Acetobacter pasteurianus* (UMCC 2951) strains performances in producing acetic acid

**DOI:** 10.3389/fmicb.2022.991688

**Published:** 2022-11-17

**Authors:** Warawut Krusong, Salvatore La China, Ruttipron Pothimon, Maria Gullo

**Affiliations:** ^1^Laboratory of Fermentation Technology, Division of Food Industrial Fermentation, School of Food Industry, King Mongkut’s Institute of Technology Ladkrabang, Bangkok, Thailand; ^2^Department of Life Sciences, University of Modena and Reggio Emilia, Reggio Emilia, Italy

**Keywords:** spore-forming bacteria, acetic acid bacteria, *Paenibacillus azoreducens*, *Acetobacter pasteurianus*, acetic acid, saccharified rice

## Abstract

In this study, spore-forming bacteria isolated from saccharified rice were selected for producing acetic acid. From the screening of 15 strains, P8 strain was chosen as a candidate. The strain was identified as *Paenibacillus azoreducens* by 16S rRNA analysis (99.85% similarity with *P. azoreducens* CM1^T^). Acetic acid is the main component of vinegar but also an industrial commodity produced by chemical synthesis. Sustainable routes for obtaining acetic acid are of great interest for decreasing the environmental impact generated by chemical syntheses. Biological acetic acid production is effective for vinegar production by acetic acid bacteria, but it cannot economically compete with the chemical synthesis for producing it as a pure commodity. Considering the need to improve the yield of pure acetic acid produced by microbial conversions, in this study, P8 strain was chosen for designing processes in different fermentation conditions. Tests were conducted in single and semi-continuous systems, using rice wine as substrate. Acetic acid produced by P8 strain was compared with that of *Acetobacter pasteurianus* (UMCC 2951), a strain known for producing acetic acid from rice wine. Even though the fermentation performances of *P. azoreducens* P8 were slightly lower than those of acetic acid bacteria usually used for vinegar production, results highlight its suitability for producing acetic acid. The final acetic acid produced by *P. azoreducens* P8 was 73 g/L, in a single stage fermentation, without losses. In nine cycles of semi-continuous regime the average of acetification rate was 0.814 (g/L/days). Two main attributes of *P. azoreducens* P8 are of relevance for producing acetic acid, namely the ability to grow at temperature higher (+ 37°C), than mesophilic acetic acid bacteria, and the absence of cytoplasmic assimilation of acetic acid. These features allow to design multiple strains cultures, in which *P. azoreducens* can acts as a helper strain. Based on our results, the new isolate *P. azoreducens* P8 can be propagated in fermenting broths for boosting acetic acid production, under the selected conditions, and used in combination with acetic acid bacteria to produce biological acetic acid, as a non-food grade commodity.

## Introduction

Spore-forming bacteria are receiving great attention for their biotechnological potential in different industrial areas and sustainable productions. Their ability to survive in different environments at high physiological stresses, make them suitable for several bioprocesses, leading to high production of useful bio-compounds (Lal and Tabacchioni, 2009; Grady et al., 2016; Kaziūnienė et al., 2022).

Spore-forming bacteria were longed classified into two orders represented by *Bacillales* and *Clostridiales* (Abecasis et al., 2013). More recently, the new order *Thermoanaerobacterales* was proposed (Wiegel, 2015). Food spoilage *Bacillales* members belong to the genera of *Bacillus*, *Geobacillus*, *Anoxybacillus*, *Alicyclobacillus* and *Paenibacillus* (Mcclure, 2006).

Among these genera, *Paenibacillus* includes Gram-positive or variable, spore-forming bacteria, rod-shaped, aerobic or facultative anaerobic. Species belonging to *Paenibacillus* were reported to be ubiquitous, widely isolated from various environment, as cow feces (Velazquez et al., 2004), plant roots and food (Berge et al., 2002), warm springs (Chou et al., 2007), raw and heat-treated milk (Scheldeman et al., 2004), paper mill white water (Chiellini et al., 2014), blood cultures (Roux and Raoult, 2004) and traditional Chinese vinegar produced from cereals (Li et al., 2016). Many species of *Paenibacillus* produce antimicrobial compounds that are useful in medicine or as pesticides, and many yield enzymes that could be utilized for bioremediation or to produce valuable chemicals (Grady et al., 2016; da Costa et al., 2022). Due to their antibacterial activity, *Paenibacillus* species were also reported to be suitable for foods and beverages production (Marwoto et al., 2004; Grady et al., 2016; Li et al., 2016). Among recognized species, *Paenibacillus azoreducens*, a facultative anaerobic bacterium able to decolorize azo dyes, grows in a temperature range between 10°C and 50°C, with optimal growth at 37°C and can produces acids from sugars (Meehan et al., 2001).

Chemically synthesized acetic acid and its derivates are commodities that have become a major feedstock for the United States and worldwide chemical industry. Petrochemically produced acetic acid reached a level of 4.68 × 109 lbs./year in the United States by 1995 and was ranked 35th in abundance of all chemicals produced (Kirschner, 1996). Worldwide production in 1998 was estimated at 11.9 × 109 lbs./year (Layman, 1998). From more recent data, the global demand for virgin acetic acid is estimated to be 16.1 million tons in 2020, and it is expected to reach 19.6 million tons by 2027 (Martín-Espejo et al., 2022). Although many research efforts, bioderived acetic acid does not compete economically with acetic acid produced by chemical synthesis. The chemical process, although is expensive and depends on non-renewable petroleum for raw materials, takes advantage of an initially high acetic acid concentration (35%–45%) and high production yield. Instead, acetic acid produced by microbial processes, *via* ethanol oxidation by acetic acid bacteria (AAB) or by anaerobic fermentation, can be obtained from a number of renewable raw materials, but the major disadvantage is the cost of recovery of low concentrations of acetic acid (4%–12%) from the fermentation broths. Moreover, during AAB fermentation other compounds, such as bacterial cellulose and gluconic acid can be formed, which could affect the final acetic acid content (La China et al., 2021; He et al., 2022). On the other hands, considering the use of vinegar in non-food productions, for instance, it can be added to correct pH in detergents preparations, together with its derivatives, acetic acid is applied in pharmaceuticals, plasticizers, solvents, textiles, heat transfer liquids, neutralizer, fungicide and de-icers productions (Kersters et al., 2006). Considering the need of the biotechnology industry in producing compounds, such as organic acid by microbial conversions, the production of acetic acid, traditionally associated to vinegar production by AAB (La China et al., 2018), could be improved in terms of yield. Moreover, due to the global demand for acetic acid, sustainable routes for obtaining it are an open challenge, as an alternative to the chemical synthesis.

In this study, we present a new strain (P8) belonging to the genus *Paenibacillus* isolated from upland rice during the saccharification process, identified as *P. azoreducens* species. Given the continuous increasing interest in members belonging to this genus, the phenotypic traits of this new strain were studied, including the ability to produce acetic acid and other volatile organic compounds (VOCs). The acetic acid production by *P. azoreducens* (P8 strain) was compared with that of *Acetobacter pasteurianus* UMCC 2951, an AAB strain previously isolated from rotten pineapple pomace and studied for its attitude in performing ethanol oxidation into acetic acid. The output of this study can be further exploited for design a multiple microbial culture composed by the two strains aimed at producing bioderived acetic acid.

## Materials and methods

### Strains isolation and cultivation conditions

Isolation of spore-forming bacteria was conducted from 30 samples of contaminated or rotten saccharified rice. Samples were diluted in peptone water (1% w/v) and aliquots (1 ml) were plated in sterile *Paenibacillus*-basal agar medium consisting of (g/L of water) glucose 20, yeast extract 5, tryptone 5, (NH_4_)_2_HPO_4_ 0.5, MgSO_4_.7H_2_O 0.25, CaCO_3_ 10 and agar 15 (modified from Nakashimada et al., 1998). Plates were incubated at 37°C for 3–5 days, under aerobic conditions. Then, single colonies were selected based on the clear halo formed on the agar medium due to acid production. Selected colonies were picked up and sub-cultured on the isolation medium until pure cultures were obtained. All isolates were checked for spore production by spore staining test, according to the procedure reported by Oktari et al. (2017). Briefly, samples of cell culture were stained by using Malachite Green solution of 5 and 0.5% Safranin. After first staining with Malachite Green by a moist heating process for 10 min, the cell culture was washed with water and then covered with paint Safranin which results in the green coloring on the spores, as well as red in the vegetative cells. The spore former-acid producing strains were named as “P” followed by a progressive number and preserved in *Paenibacillus*-basal medium agar slant, at 4°C.

### Selection of spore former-acid producing bacteria and acetic acid production tests

Upland rice vinegar, containing 80 g/L of acetic acid was obtained from the Laboratory of Fermentation Technology, King Mongkut’s Institute of Technology Ladkrabang, Thailand. It was used as a substrate in acetification medium. Meanwhile, upland rice wine with an ethanol content of 90 ± 0.2 g/L and titratable acidity of 1.8 ± 0.2 g/L was also used in the adjustment of ethanol in acetification medium. All spore former-acid producing strains were cultivated in acetification medium consisting of (g/L of water) glucose 50, yeast extract 5, MgSO_4_.7H_2_O 0.2 and (NH_4_)_2_HPO_4_ 0.5 which was adjusted to a total concentration (TC; a parameter which expresses the maximal concentration of acetic acid that can be obtained in a complete fermentation) of 80 g/L by using upland rice wine and vinegar in a 1 l Duran bottle (working volume 500 ml), as described by Krusong et al. (2014, 2015). The aeration was controlled at a constant rate of 4 l/min.

Upland rice vinegar and wine were used for adjustment of acetic acid and ethanol contents in the medium of acetification process by P8 and *A. pasteurianus* UMCC 2951 strains, respectively. *A. pasteurianus* UMCC 2951 strain was previously adapted to high acetic acid concentration at 30 ± 1°C by stepwise scaling up, starting from 10 g/L to 65 ± 1 g/L of acetic acid, over a six-year period (Krusong and Tantratian, 2014; Pothimon et al., 2020). The broth culture from *A. pasteurianus* UMCC 2951 strain was prepared by using the following medium (g/L of water): glucose 50, yeast extract 5, MgSO_4_.7H_2_O 0.2 and (NH_4_)_2_HPO_4_ 0.5, under aeration (4.5 L/min), for 7 days at 30 ± 1°C (Krusong et al., 2007). According to the high initial acetic acid concentration (HAA_i_) process previously set up (Krusong et al., 2014), the acetification medium (g/L of water: glucose 50, yeast extract 5, MgSO_4_.7H_2_O 0.2 and (NH_4_)_2_HPO_4_ 0.5) was standardized to a total concentration of 80 g/L, by adjusting the ethanol and acetic acid contents to 35 ± 1 g/L and 45 ± 1 g/L, respectively.

### 16S rRNA gene sequencing and phylogenetic analysis

Strain identity was determined by 16S rRNA gene sequencing. Genomic DNA extraction, amplification and sequencing of 16S rRNA gene was performed by Macrogen Inc. company, according to their protocols. Then, the sequence was trimmed, removing low quality bases, using Phred v 0.071220.c and assembled using Pharp v 1.090518 (Ewing and Green, 1998). The dataset was structured by downloading a total of 20 16S rRNA sequences of *Paenibacillus* strains from NCBI 16S rRNA database, selecting sequencing of strains isolated from food matrices. The 16S rRNA sequences were aligned using Muscle v3.8.31 (Edgar, 2004). Resulting alignment was imported in MegaX (Kumar et al., 2018) and trimmed in to obtain sequences with the same length. Trimmed alignment was used to generate a maximum-likelihood (ML) phylogenetic tree, applying the Tamura-Nei evolutionary model (Tamura and Nei, 1993), setting a discrete gamma distribution to model evolutionary rate differences among sites. The ML phylogenetic tree was computed using 1,000 replicates. The alignment was also used to calculate the phylogenetic distant matrix. In addition, the nucleotide sequence of 16S rRNA gene is available at GenBank, under the accession number OP353700.1.

### Acetification performance by single and semi-continuous processes

The acetification conditions (single stage) for both P8 and *A. pasteurianus* UMCC 2951 strains were setup in a 100 L internal Venturi injector bioreactor (as reported in our previous study; Krusong et al., 2015), which comprised a stainless-steel tank 1.00 m high and 0.40 m internal diameter that had a maximum working volume of 75 l. The medium (consisting of (g/L of water): glucose 50, yeast extract 5, MgSO_4_.7H_2_O 0.2 and (NH_4_)_2_HPO_4_ 0.5 which was adjusted to a TC of 80 g/L by using upland rice wine and vinegar) was recycled using a centrifugal pump (Grundfos Ltd., Bangkok, Thailand), filtered and entrained. Well-compressed air was introduced into the medium at the injector nozzle (Mazzei Injector Com., LLC, Bakersfield, CA, USA) at 7.25–14.5 psi creating a plentiful amount of fine air bubbles in the bioreactor. The temperature of the medium was controlled at 30°C by cooling unit with Thermocouple Resistance Temperature Detector (RTD) PT100 RTD (Omega Engineering Inc., Connecticut, USA).

The startup phase was conducted with 25 L of working volume of the bioreactor at 7.25 psi of air (Krusong et al., 2015). The end of this phase occurred when the ethanol content reached 5 g/L or less (Fregapane et al., 2001; de Ory et al., 2004). Then, the fermentation phase was started by adding a volume of fresh medium to make up the medium volume to 75 L at 14.5 psi of air (Krusong et al., 2015), fixing TC at 80 ± 1 g/L. The overoxidation of acetic acid was tested after the ethanol content reached 0 g/L, by extending acetification period under the same rate of aeration supply. Samples were collected at the end of the operational phase.

The semi-continuous acetification by P8 strain under HAA_i_ conditions was conducted fixing TC at 80 g/L (consisting of 45 g/L acetic acid and 35 g/L ethanol) in the 100 l internal Venturi injector bioreactor. The temperature was controlled at 30°C. Acetification rate (ETA), which measures the rate of acetic acid production (difference between the final and initial acidity during each acetification cycle) and biotransformation yield (percentage of ethanol that is converted to acetic acid; Fregapane et al., 2001; de Ory et al., 2004; Krusong et al., 2015) were used as indicators of process effectiveness. The means of the calculated values of these two parameters were recorded for each cycle, along with the cell biomass in forms of cell dried weight (CDW) values.

### Analytical determinations

Acetic acid and ethanol content during acetification was measured using GC–MS (Thermo Scientific Trace GC Ultra coupled to an ISQ Single Quadrupole Mass Spectrometer, Thermo Fisher Scientific Inc., Waltham, Massachusetts, USA). The DB-wax column (length 30 m, Pressure 6.76 psi, Flow 1.0 ml/min) was used with an inlet temperature of 250°C and with splitless injection of 75 ml/min. Helium gas was employed as the carrier at 1.2 ml/min. Samples were introduced and maintained at 40°C for 5 min. The temperature increment was 5°C/min to 120°C for acetic acid or 5°C/min to 90°C for ethanol, and this was then held constant for 10 min and 3 min, respectively. Identification of acetic acid or ethanol was based on retention times compared with the Wiley, 275.L data library for the GC–MS system. Standard curve of acetic acid and ethanol was carried out and used for concentration evaluation. The cell biomass in terms of CDW in each acetification cycle was measured from the absorbance at 660 nm with a spectrophotometer (GENESYS 10VIS). The sample was diluted to an OD660nm value between 0.3 and 0.8 and converted to CDW through a linear correlation standard curve of *P. azoreducens,* one DO660 was almost equivalent to 0.3 g/L. The resulting CDW was determined in the same way as that in the fermentation medium (Krusong and Tantratian, 2014).

The volatile compounds (VOCs) produced by P8 and *A. pasteurianus* UMCC 2951 strains were removed by solid phase micro-extraction (SPME; modified from Vas and Vékey, 2004) and measured using GC–MS. First, 5 ml of the sample was placed in a 25 ml glass bottle leaving a 20 ml headspace volume. Then, 3 g of solid NaCl was added and the bottle was sealed with a septum cap (Stableflex PDMS/DVB for SPME fiber size 60 μm; Supelco Inc., Bellefonte, PA, USA). After 60 min at 37°C, to allow for extraction, the components were analyzed using GC–MS (as above). Samples were introduced and maintained at 40°C for 5 min. The temperature increment was 5°C/min to 230°C and was then held constant for 5 min. For MS determination, an electron ionization mode was employed with ion source temperature of 230°C, scan mass range of 35–300 amu, and MS transfer line at 240°C with 0 min solvent delay time. Identification of the volatile components was based on retention times and mass spectra fragmentation patterns and qualitatively compared with the Wiley, 275.L data library for the GC–MS system.

### Statistical analyses

Measurements were replicated three times and their means are reported together with appropriate standard deviations (±standard deviation). The statistical significance was determined using one-way ANOVA and differences among samples were assessed using Tukey *post-hoc* test, when required. All statistical tests were performed using R, Version 4.1.0.

## Results and discussion

### Isolation and selection of spore former-acid producing bacteria from saccharified rice

**Figure 1:**
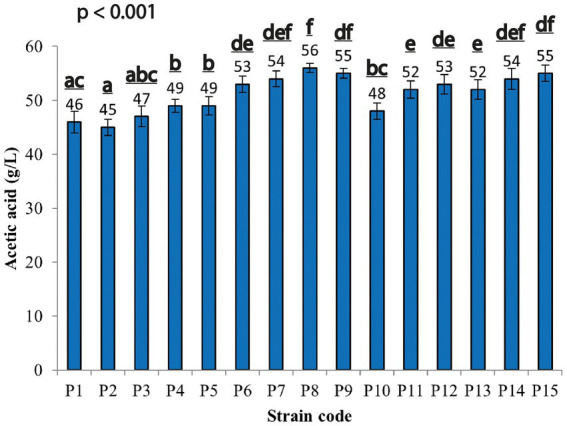
Acetic acid production by spore former-acid producing strains at 30 ± 2°C during 30 days of cultivation. Each value is the mean of three parallel replicates ± standard deviation. Significant differences among the samples in acetic acid production were represented by different letters.

In this study, 60 isolates were collected from a total of 30 samples of contaminated saccharified rice. Fifteen strains (labeled from P1 to P15) produced both spores and a clear halo area around the colony on *Paenibacillus*-basal agar medium supplemented with CaCO_3_, indicating that they were acid producers. To evaluate the acetic acid ability of the spore forming strains, tests were conducted in Duran bottles for 30 days, at 30 ± 2°C; TC: 80 g/L (45 g/L acetic acid and 35 g/L ethanol, respectively), and aeration at 4 l/min, according to the method described by Krusong et al., 2015. All the strains produced acetic acid in the range between 45–55 g/L, except for strain P8, which produced a higher acetic acid amount (56 g/L; Figure 1). Given the highest amount of acetic acid by P8 strain, it was selected for further investigation.

### Species level identification of P8 strain

**Figure 2:**
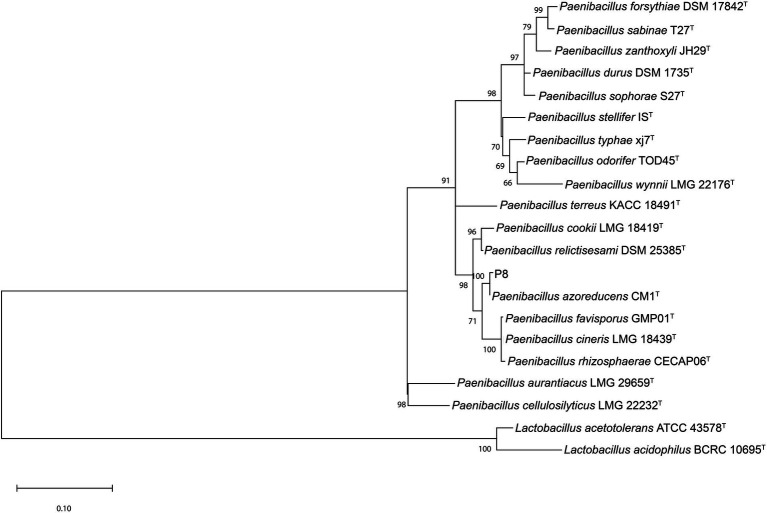
Phylogenetic tree of based on the 16S rRNA gene sequence of strain P8 and representative strains from NCBI.

An almost complete 16S rRNA gene sequence (1,515 bp) from gDNA of P8 strain was determined. Sequences, from the multiple alignment of the 20 sequences retrieved from NCBI and P8 16S rRNA sequence were trimmed to the same length, reducing the total sequence length to 1,354. The ML phylogenetic tree obtained (Figure 2) showed the presence of three major clades. 16S rRNA sequence of P8 strain had highest similarity (99.85%) with *P. azoreducens* CM1^T^. The bootstrap value of 100, highlights the strength of the analysis. The distant matrix (Supplementary material) computed, excluding the outgroup represented by *Lactobacillus* strains, showed that the distant index of both P8 and *P. azoreducens* CM1^T^ was 0.145%, meaning the sequence similarity was 99.85%. The average distant values among overall strains were calculated resulting in 5.44%. Given the high similarity percentage compared with the average distance calculated for all 21 strains, it can be assumed that P8 is a new strain of *P. azoreducens* species. Bacteria belonging to *Paenibacillus* are widely distributed in natural matrices, especially in soil and plant roots (Grady et al., 2016). *P. azoreduncens* strains were isolated from textile wastewater and were recognized as synthetic azo dye producers (Meehan et al., 2001).

### Performance of *Paenibacillus azoreducens* P8 and *Acetobacter pasteurianus* UMCC 2951 in producing acetic acid

**Figure 3:**
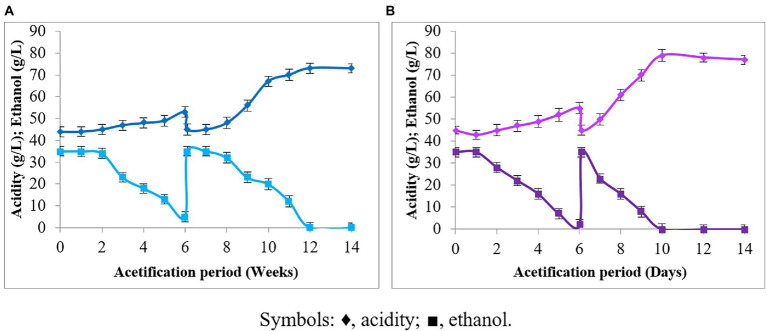
Acetification under HAA_i_ conditions: **(A)** *Paenibacillus azoreducens* P8; **(B)** *A. pasteurianus* (UMCC 2951). Acetic acid and ethanol concentrations were adjusted constantly at 45 and 35 g/L, respectively. Symbols: ♦, acidity (in terms of acetic acid); ■, ethanol. Each value is the mean of three parallel replicates ± standard deviation.

The production of acetic acid of *P. azoreducens* P8 strain in scaling up conditions was first tested in Venturi injector bioreactor and results compared with those obtained from *A. pasteurianus* UMCC 2951, which is a well described strain for its aptitude to produce acetic acid under environmental stressors, like high temperature and HAA_i_ (Krusong and Tantratian, 2014; Pothimon et al., 2020). The results obtained showed that *P. azoreducens* P8 strain was able to start ethanol oxidization after 2 weeks of cultivation (Figure 3A). During the second cycle, at 6 weeks, the ethanol oxidation started after 1 week of the fermentation process. Finally, after 12 days of fermentation, the ethanol was completely oxidized, reaching a final acetic acid content of 73 g/L. Considering *A. pasteurianus* UMCC 2951, our data highlighted that the trends were almost the same comparing to P8 strain (Figure 3B). About the fermentation process for *A. pasteurianus* UMCC 2951, the start-up period was shorter. Indeed, the ethanol fermentation started after 1 day of cultivation and the first cycle finished at day 6. The second cycle was started on day 6 and after 4 days the ethanol was completely oxidized into acetic acid. However, it could be noticed that the same characteristics of acetification profile consisting of start-up and operational phases of both cultures were similar, but only difference in acetification period was observed. The difference in acetic acid production by *P. azoreducens* P8 and *A. pasteurianus* UMCC 2951 can be explained considering the different cultivation history of the two strains. *A. pasteurianus* UMCC 2951 was subjected to a selective pressure along 6 years of adaptation at high acetic acid content and high temperature (Pothimon et al., 2020). Instead, P8 is a new isolated strain. The propagation of microbial cultures under selective pressure, is well known in the case of AAB generally used for industrial vinegar production, where cultures acquire resistance to environmental stressors if continuously maintained under stringent physiological conditions (Azuma et al., 2009; Gullo et al., 2012).

### Semi-continuous acetification by *Paenibacillus azoreducens* P8

To evaluate large scale production of acetic acid by *P. azoreducens* P8, a semi-continuous process, in a 100 L internal Venturi injector bioreactor, was developed. As reported by many studies the biological process of vinegar fermentation can be considered completed when the amount of residual ethanol is around or below 5 g/L (Ebner et al., 1996; Ndoye et al., 2007; Gullo et al., 2014; Krusong et al., 2015). In this study, between each cycle, a 40% of the total volume was replaced by fresh medium and, subsequently, the new cycle was started. A total of nine cycles were performed in which *P. azoreducens* P8 was continuously under stressors, like high acetic acid and ethanol. Full data are reported in Table 1. In the startup phase and the first cycle, the ETA values (0.214 g/L/d and 0.671 g/L/d, respectively) were low, showing an initial adaptation stage of the culture to fermentation. This consideration is supported by the CDW values, showing that the weight of the dried cell was 0.026 at the start-up phase and 0.029 at cycle 1. However, during the first cycle, the ETA value reached 0.671 g/L/d, indicating the fermentation start. From cycle 2 until the end of the process, the amount of acetic acid produced was considerable (about 30 g/L for each cycle) except for cycles 6 and 7 in which a slight decrease was observed (24 ± 0.3 g/L and 25 ± 0.3 g/L, respectively). This reduction can be due to the oscillation of the cell viability (CDW 0.027 in the cycle 6 and 0.031 in the cycle 7), that generally might occur (Maestre et al., 2008) considering the toxic effect of acetic acid on cells (Saeki et al., 1997; Lu et al., 1999; Baena-Ruano et al., 2010; Song et al., 2022). The ETA and the CDW values were consistently with the acetic acid production trend. The biotransformation percentage, excluding the startup phase, ranged from 75% up to 97% (90.1% as average). Comparing *P. azoreducens* P8 with *A. pasteurianus* UMCC 2951, previously tested (Pothimon et al., 2020), the average of acetic acid production was slightly reduced (30.11 g/L on average of acetic acid produced by P8; 52 g/L on average of *A. pasteurianus* UMCC 2951). Compared to others AAB used in semi-continuous processes, the ETA values resulted to be low, such as 2.2 + 0.06 g/L/d by an industrial culture of vinegar (Fregapane et al., 2001); 12 g/L/d by *A. senegalensis* (CWBI-B418^T^) (Ndoye et al., 2007) and 5 g/L/h *A. pasteurianus* (Mounir et al., 2018). These differences can be correlated to the handling of cultures, especially to the different cultivation time under selective pressure, as previously discussed (Paragraph 3.3). Thus, the new isolate *P. azoreducens* P8, which showed appreciable aptitude to produce acetic acid, can be considered a suitable candidate for industrial acetic acid production. It is reasonable to suppose that longer adaptation cycles to fermentation conditions could further enhance the amount of acetic acid produced.

**Table 1:** Semi-continuous acetification under HAA_i_ conditions by *Paenibacillus azoreducens* P8: 45 + 1 g/L acetic acid with constant 35 + 1 g/L ethanol concentration at 30 + 1°C (TC 80 g/L).

	Experiment time(week)	Total acidity^a^		Aceti acid produced(g/L)	ETA(g/L/d)	Ethanol^a^		Ethanol oxidized^a^(g/L)	Transformation yield(%)	CDW(g/L)
		Initial (g/L)	Final (g/L)			Initial (g/L)	Final (g/L)			
Start up	6	44 ± 0.2	53 ± 0.1	9 ± 0.1	0.214	35 ± 0.1	4.9 ± 0.1	30.1 ± 0.1	29.9	0.026
Cycle 1	6	45 ± 0.3	73 ± 0.3	28 ± 0.3	0.671	35 ± 0.3	3 ± 0.2	32 ± 0.2	87.5	0.029
Cycle 2	5	45 ± 0.2	75 ± 0.2	30 ± 0.2	0.857	35 ± 0.4	1 ± 0.1	34 ± 0.2	88.2	0.043
Cycle 3	5	45 ± 0.3	78 ± 0.2	33 ± 0.2	0.943	35 ± 0.1	1 ± 0.1	34 ± 0.1	97.1	0.045
Cycle 4	5	45 ± 0.2	76 ± 0.3	31 ± 0.2	0.886	35 ± 0.3	2 ± 0.3	33 ± 0.3	93.9	0.04
Cycle 5	5	45 ± 0.5	78 ± 0.4	33 ± 0.4	0.943	35 ± 0.3	1 ± 0.2	34 ± 0.2	97.1	0.048
Cycle 6	6	45 ± 0.2	69 ± 0.5	24 ± 0.3	0.571	35 ± 0.1	3 ± 0.1	32 ± 0.1	75	0.027
Cycle 7	6	45 ± 0.4	70 ± 0.2	25 ± 0.3	0.6	35 ± 0.1	3 ± 0.1	32 ± 0.1	78.1	0.031
Cycle 8	5	45 ± 0.5	78 ± 0.2	34 ± 0.3	0.971	35 ± 0.3	0	35 ± 0.3	97.1	0.042
Cycle 9	5	45 ± 0.3	78 ± 0.5	33 ± 0.4	0.943	35 ± 0.2	1 ± 0.1	34 ± 0.1	97.1	0.048
Average^b^					**0.814**				**90.1**	

a Values represent the mean ± standard deviation of triplicate.

b Average ETA and transformation yield (values in bold) were calculated among nine semi-continuous cycles, not including start-up phase.

ETA, acetification rate; CDW, cell dried weight.

At current this strategy is not aimed at producing food grade acetic acid because the absence of deep information on the use of *Paenibacillus* strains in food, but it can be of great relevance to produce biological acetic acid for non-food uses.

Main advantages of the strategy proposed in this study arise at least from two technological traits that can affect the biological production of acetic acid. AAB have optimal temperature growth of 28–30°C and in absence of thermotolerant strains, the oxidation of ethanol into acetic acid could be stopped because of the exergonic nature of the reaction (Ebner et al., 1996; Gullo et al., 2016). Moreover, AAB could oxidize acetic acid when ethanol is depleted and can produce bacterial cellulose, which is undesired during organic acid production (La China et al., 2018). If the process is aimed at producing acetic acid at higher rate than vinegar, a multiple culture of AAB and other bacteria, which can growth at higher temperature and that do not oxidize acetic acid could be an innovative introduction for the biological production of acetic acid. Based on these considerations *P. azoreducens* P8 can be considered a helper strain in producing acetic acid in a multiple starter, as a booster of the fermentation, contributing to maintain the acetic acid level, avoiding losses during processes.

### Volatile compounds produced by *Paenibacillus azoreducens* P8 and *Acetobacter pasteurianus* UMCC 2951 strains, under HAA_i_ conditions

In this study, to have more knowledge on microbial compounds of industrial interest from *P. azoreducens* P8 and *A. pasteurianus* UMCC 2951, VOCs composition was determined. The VOCs composition of rice wine, which was the alcoholic substrate used to produce acetic acid, is highly complex and depends on many factors, including the microbial composition of the starter and the fermentation practices. Main VOCs categories, previously described, belong to esters, alcohols, amino acids, and organic acids (Zhang et al., 2022).

**Figure 4:**
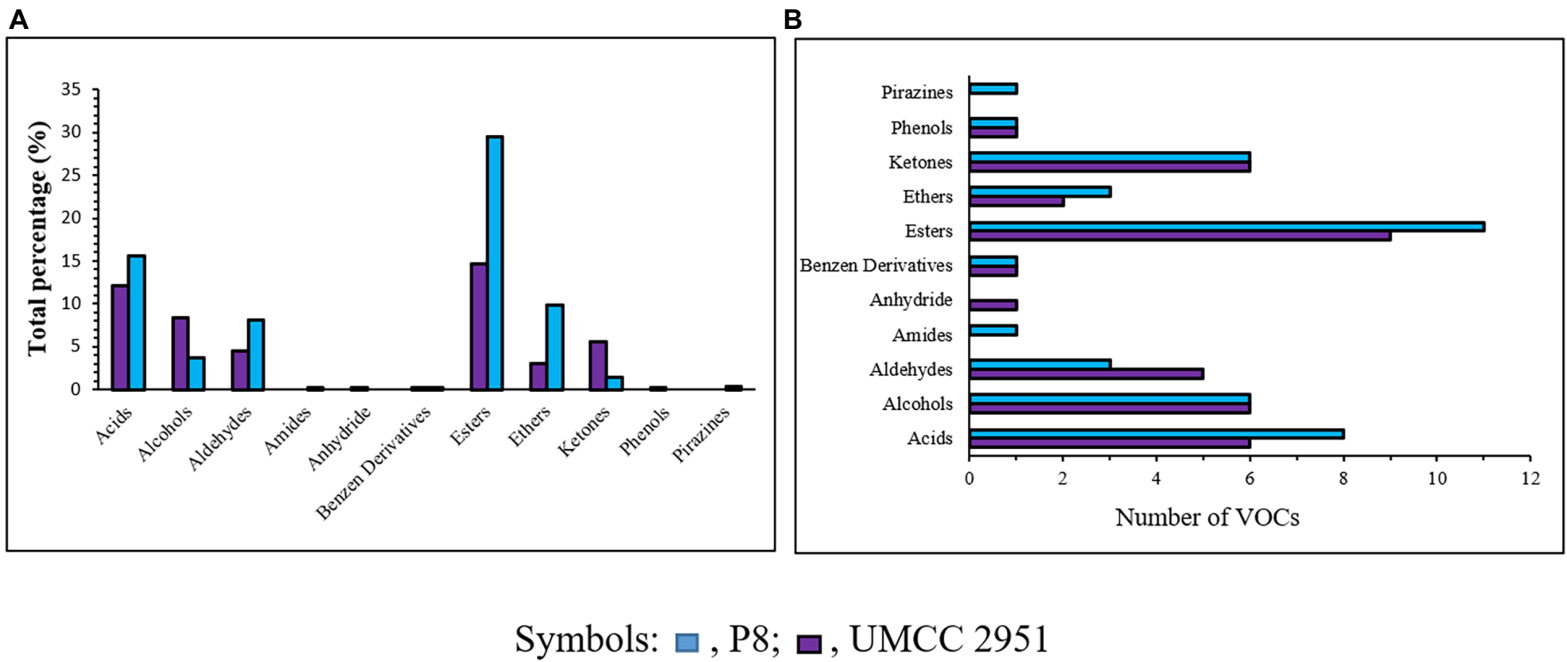
VOCs classification in the fermentation process using both *P. azoreducens* P8 and *Acetobacter pasteurianus* (UMCC 2951). **(A)** Number of compounds grouped in chemical families. **(B)** Total percentages for each chemical family detected.

The VOCs distribution in terms of chemical families was found to be different among the fermented products obtained by *P. azoreducens* P8 and *A. pasteurianus* UMCC 2951, respectively (Figure 4A). A total of 40 and 37 VOCs were found in *P. azoreducens* P8 and *A. pasteurianus* UMCC 2951 fermented products, respectively. Among all the identified VOCs, 20 were produced by both culture (Table 2). The most abundant VOCs belonged to esters (29.46% for P8 and 14.62% for UMCC 2951) and acids (15.54% for P8 and 12.14 for UMCC 2951; Figure 4B). VOCs in these chemical families were represented by products or intermediates of the oxidation of ethanol into acetic acid. In the case of esters, the most abundant VOC was ethyl acetate for both culture strains, whereas for acids, the most abundant VOC was acetic acid. Other VOCs species belong to these chemical families were intermediates of the fermentation process (e.g., 2-methyl propanoic acid or pentanoic acid) detected in different amount and different chemical species. Regarding other abundant chemical families, alcohols and aldehydes were the most abundant. In detail, we observed the same number of species of alcohols in both cultures (6 per each sample), but considering the total percentage, alcohols were most abundant in *A. pasteurianus* UMCC 2951 (8.41%) culture compared to *P. azoreducens* P8 (3.72%). In the case of aldehydes, the number of VOCs were different (3 for P8 and 5 for *A. pasteurianus* UMCC 2951, while the total percentage was higher in P8 culture (8.1%) compared to *A. pasteurianus* UMCC 2951 (4.46%). Among these chemical families, the two samples were characterized by a different amount of VOCs belonging to acids and esters. In particular, in *P. azoreducens* P8 culture the number of acids and esters was higher than in *A. pasteurianus* UMCC 2951 culture. Also, ethers were found to be abundant in both samples, (9.8% of the total in P8 and 2.98% in UMCC 2951; Figure 4B). In addition to the chemical families discussed, other VOCs belong to different chemical families were detected in small amount. Phenols and benzene derivatives were detected in both samples (Figure 4A). Some chemical species were detected just in one sample. For example, one amide (3-amino-4,5,6-trimethyl-thieno[2,3-b]pyridine-2-carboxylic acid tert-butylamide) and one pyrazine (methyl-pyrazine) were produced by *P. azoreducens* P8. The VOC analysis performed in this study provided data of interest for further studies aimed at building up a coculture fermentation for evaluating the amount of acetic acid and other microbial products of industrial interest.

**Table 2:** VOCs composition in samples obtained from *P. azoreducens* P8 and *A. aceti* WK acetification.

CAS#	Chemical family	Formula	*P. azoreducens* P8			*Acetobacter pasteurianus* UMCC 2951		
			% of total	Component area	Component RT	% of Total	Component area	Component RT
	Acids							
3,724-65-0	2-Butenoic acid	C_4_H_6_O_2_	-	-	-	0.04	5065418.9	25.3566
64-19-7	*Acetic acid*	*C_2_H_4_O_2_*	7.21	5,789,197,422	18.3692	7.89	7,142,863,733	18.2338
65-85-0	Benzoic acid	C_7_H_6_O_2_	0.05	7,451,135.9	33.8314	-	-	-
107-92-6	*Butanoic acid*	*C_4_H_8_O_2_*	0.45	67,991,028.8	22.9552	0.21	25,357,116.2	22.9643
334-48-5	Decanoic acid	C_10_H_20_O_2_	0.01	1,056,726.4	31.8485	-	-	-
142-62-1	*Hexanoic acid*	*C_6_H_12_O_2_*	0.53	79,128,285.9	26.3835	0.08	9,829,193	26.3913
124-07-2	Octanoic acid	C_8_H_16_O_2_	0.26	39,195,729.5	29.2712	-	-	-
109-52-4	*Pentanoic acid*	*C_5_H_10_O_2_*	5.02	752,384,101	23.6423	1.8	216,205,301	23.6736
79-31-2	*Propanoic acid, 2-methyl-*	*C_4_H_8_O_2_*	2.01	301,178,116	21.8405	2.12	253,806,031	21.8385
	Alcohols							
24,629-25-2	(S)-(+)-Isoleucinol	C_6_H_15_NO	0.11	16,888,981.6	25.3551	-	-	-
60-12-8	*Benzeneethanol*	*C_8_H_10_O*	2.32	348,606,611	27.436	-	-	-
100-51-6	Benzyl alcohol	C_7_H_8_O	-	-	-	0.06	7,094,432.2	26.9161
913,176-41-7	Caryophyllenyl alcohol	C_15_H_26_O	0.02	2,530,766.1	29.4892	-	-	-
123-51-3	*Isoamylalcohol*	*C_5_H_12_O*	1.24	185,441,662	13.3035	2.91	348,946,524	13.4474
78-83-1	*Isobutylalcohol*	*C_4_H_10_O*	0.02	2,559,288.9	9.6436	0.07	8,118,441.1	9.6442
10,482-56-1	L-.alpha.-Terpineol	C_10_H_18_O	-	-	-	0.03	3,331,502.1	24.2235
108-95-2	Phenol	C_6_H_6_O	0.01	1,694,766.4	28.6518	-	-	-
	Aldehydes							
98-01-1	2-furan-carboxaldehyde	C_5_H_4_O_2_	4.47	670,371,399	19.8976	-	-	-
620-02-0	2-Furancarboxaldehyde, 5-methyl-	C_6_H_6_O_2_	-	-	-	0.46	55,092,770.7	22.1427
67-47-0	5-Hydroxymethylfurfural	C_6_H_6_O_3_	-	-	-	0.01	1,561,246.1	34.651
75-07-0	*Acetaldehyde*	*C_2_H_4_O*	0.03	4,828,477.5	3.4066	0.06	6,999,246.9	3.4061
100-52-7	*Benzaldehyde*	*C_7_H_6_O*	3.6	540,672,024	21.2164	3.86	463,180,656	21.2608
122-78-1	Phenyleacetaldehyde	C_8_H_8_O	-	-	-	0.07	8,709,543.5	23.4808
	Esters							
2,000,696-25-7	.beta.-1,5-O-Dibenzoyl-ribofuranose	C_19_H_18_O_7_	-	-	-	0.03	3,794,906.6	33.8524
108-64-5	1-Butanol, 3-methyl-, acetate	C_7_H_14_O_2_	0.16	24,353,399.3	9.0075	-	-	-
123-92-2	*1-Butanol, 3-methyl-, acetate*	*C_7_H_14_O_2_*	0.23	34,999,276.3	10.7144	0.83	98,998,845.5	10.8224
96-48-0	2(3H)-Furanone, dihydro-	C_4_H_6_O_2_	-	-	-	0.07	7,850,092.2	23.235
7,779-72-8	3-methylbutyl 2-oxopropanoate	C_8_H_14_O_3_	-	-	-	0.22	25,893,946.9	10.5573
2,000,224-92-1	5,6,7,8-Tetrahydro-8,8-dimethyl-2-indolizinecarboxylic acid methyl ester	C_12_H_17_NO_2_	11	1,650,383,131	32.2686	-	-	-
110-19-0	Acetic acid, 2-methylpropyl ester	C_6_H_12_O_2_	0.93	139,127,424	7.5917	-	-	-
103-45-7	*Acetic acid, 2-phenylethyl ester*	*C_10_H_12_O_2_*	0.41	61,756,862.5	26.1146	0.82	98,203,150.3	26.1187
101-97-3	Acetic acid, phenyl-, ethyl ester	C_10_H_12_O_2_	0.16	23,761,288.8	25.6463	-	-	-
140-11-4	Acetic acid, phenylmethyl ester	C_9_H_10_O_2_	-	-	-	0.03	3,705,089	24.7854
2,000,532-52-8	Adipic acid, 2,4-dimethylpent-3-yl isobutyl ester	C_17_H_32_O_4_	0.02	3,406,092.8	27.0345	-	-	-
105-54-4	Butanoic acid, ethyl ester	C_6_H_12_O_2_	0.63	94,570,465.9	8.1925	-	-	-
141-78-6	*Ethyl Acetate*	*C_4_H_8_O_2_*	15.69	2,353,245,444	5.0955	10.91	1,308,584,233	5.0465
110-19-0	Isobutyl acetate	C_6_H_12_O_2_	-	-	-	1.02	122,616,009	7.5834
166,273-38-7	Pentanoic acid, 5-hydroxy-, 2,4-di-t-butylphenyl esters	C_19_H_30_O_3_	0.01	892,790.9	28.7834	-	-	-
97-64-3	*Propanoic acid, 2-hydroxy-, ethyl ester*	*C_5_H_10_O_3_*	0.22	33,654,169.2	17.04	0.69	82,300,126.9	17.0678
	Ketones							
2,000,008-43-4	1 - cyclopentene - 3,4 - di - one	C_5_H_4_O_2_	-	-	-	0.29	35,233,139.8	22.3792
56,599-61-2	1,3-Dioxolane, 4,5-dimethyl-2-pentadecyl-	C_20_H_40_O_2_	0.01	793,322.3	11.5766	-	-	-
431-03-8	2,3-Butanedione	C_4_H_6_O_2_	-	-	-	2.26	271,480,012	6.803
513-86-0	*2-Butanone, 3-hydroxy-*	*C_4_H_8_O_2_*	1.13	168,997,873	15.5356	2.39	286,712,291	15.6043
10,150-87-5	*2-Butanone, 4-(acetyloxy)-*	*C_6_H_10_O_3_*	0.17	25,995,012.6	21.7543	0.49	12,569,367.4	21.7532
4,906-24-5	*3-(Acetyloxy)-2-butanone*	*C_6_H_10_O_3_*	0.14	20,934,458.3	18.0772	0.11	13,094,275.1	18.0929
127-51-5	3-Buten-2-one, 3-methyl-4-(2,6,6-trimethyl-2-cyclohexen-1-yl)-	C_14_H_22_O	0.01	1,576,299.6	26.7035	-	-	-
98,419-10-4	4,4-Dimethyl-3-(3-methylbut-2-enylidene)octane-2,7-dione	C_15_H_24_O_2_	-	-	-	0.01	1,016,732.5	30.6742
2,715-54-0	Acetophenone, 2,4,5-triethyl-	C_14_H_20_O	0.01	2,027,351.4	25.8559	-	-	-
	Ethers							
624-92-0	*Disulfide, dimethyl*	*C_2_H_6_S_2_*	0.02	3,128,137.3	9.1197	0.09	11,247,301.7	9.1324
115-10-6	*Methane, oxybis-*	*C_2_H_6_O*	9.64	1,446,391,003	5.8865	2.89	347,279,203	5.8372
20,662-83-3	Oxazole, 4,5-dimethyl-	C_5_H_7_NO	0.14	20,468,078.4	11.9103	-	-	-
	Benzene derivatives							
91-20-3	*Naphthalene*	*C_10_H_8_*	0.02	3,087,984.8	25.1307	0.07	8,544,835.9	25.1377
	Anhydrides							
108-24-7	Acetic acid, anhydride	C_4_H_6_O_3_	-	-	-	0.09	10,738,673.1	14.2065
	Amides							
2,000,501-84-7	3-amino-4,5,6-trimethyl-thieno[2,3-B]pyridine-2-carboxylic acid tert-butylamide	C_15_H_21_N_3_OS	0.11	16,468,136.4	36.1808	-	-	-
	Phenol							
54,063-14-8	1,3-Dioxan-5-ol, 4,4,5-trimethyl-	C_7_H_14_O_3_	-	-	-	0.04	4,713,875.2	17.5423
	Pirazines							
109-08-0	Pyrazine, methyl-	C_5_H_6_N_2_	0.32	48,512,571.6	15.1769	-	-	-

VOCs retrieved were grouped based on chemical families.

## Conclusion

In study we screened and characterized a new spore-former acid producing bacterium, identified as *P. azoreducens*, strain P8. The strain was tested for the ability of producing acetic acid following industrial vinegar technology. The results obtained testing *P. azoreducens* P8 highlight the abilities of the strain in acetification process, even though the fermentation performances were slightly lower than the AAB usually used at industrial scale. *P. azoreducens* P8 possessed ethanol and acidity tolerances, developed during the early stages of HAA_i_ process (start up and the cycle 1). Comparing the fermentation activities of *P. azoreducens* P8 with *A. pasteurianus* UMCC 2951, the amount of acetic acid obtained was relatively high. Regarding the VOCs analysis, some of them were shared among the fermented broth by the *P. azoreducens* P8 and *A. pasteurianus* UMCC 2951. Given the advantages of the application in the fermentation processes at industrial of spore-forming bacteria, we can confirm that *P. azoreducens* P8, after future analysis to better clarify its genomic and phenotypic features, could be suitable for fermentation processes. Based on the output of this study, it is reasonable to suppose its use in single or multiple cultures to improve the stability and the production yield of acetic acid by biological processes.

## Data availability statement

The data presented in the study are deposited in the Genebank repository, accession number OP353700.1.

## Author contributions

WK: conceptualization, funding acquisition, project administration, validation, and writing—original draft. MG: conceptualization, validation, review and editing, and funding acquisition. RP: methodology, formal analysis, and investigation. SL: conceptualization, validation, and writing—review. All authors contributed to the article and approved the submitted version.

## Funding

The authors gratefully acknowledge two funding sources of this research consisting of School of Food Industry Research Fund (2562–01-07001), King Mongkut’s Institute of Technology Ladkrabang, Thailand and Thailand Toray Science Foundation: Science and Technology Research Grant (Year 2018).

## Conflict of interest

The authors declare that the research was conducted in the absence of any commercial or financial relationships that could be construed as a potential conflict of interest.

## Publisher’s note

All claims expressed in this article are solely those of the authors and do not necessarily represent those of their affiliated organizations, or those of the publisher, the editors and the reviewers. Any product that may be evaluated in this article, or claim that may be made by its manufacturer, is not guaranteed or endorsed by the publisher.
